# Towards User-Generalizable Wearable-Sensor-Based Human Activity Recognition: A Multi-Task Contrastive Learning Approach

**DOI:** 10.3390/s25226988

**Published:** 2025-11-15

**Authors:** Pengyu Guo, Masaya Nakayama

**Affiliations:** 1 Department of Electronic Engineering and Information Systems, The University of Tokyo, Tokyo 113-8654, Japan; kaku-houu@g.ecc.u-tokyo.ac.jp; 2 Information Technology Center, The University of Tokyo, Tokyo 113-8654, Japan

**Keywords:** Human Activity Recognition (HAR), user-generalization, contrastive learning, multi-task learning, wearable sensor, supervised contrastive learning

## Abstract

Human Activity Recognition (HAR) using wearable sensors has shown great potential for personalized health management and ubiquitous computing. However, existing deep learning-based HAR models often suffer from poor user-level generalization, which limits their deployment in real-world scenarios. In this work, we propose a novel multi-task contrastive learning framework that jointly optimizes activity classification and supervised contrastive objectives to enhance generalization across unseen users. By leveraging both activity and user labels to construct semantically meaningful contrastive pairs, our method improves representation learning while maintaining user-agnostic inference at test time. We evaluate the proposed framework on three public HAR datasets using cross-user splits, achieving comparable results to both supervised and self-supervised baselines. Extensive ablation studies further confirm the effectiveness of our design choices, including multi-task training and the integration of user-aware contrastive supervision. These results highlight the potential of our approach for building more generalizable and scalable HAR systems.

## 1. Introduction

Human Activity Recognition (HAR) is a general task that aims to recognize human activities based on various types of signals, such as images/videos, wireless signals, and wearable sensors. It plays a crucial role in a wide range of applications that directly impact health, safety, efficiency, and overall quality of life.

With the rapid development of the global economy and digital technology, the concept of “active health” has gained increasing attention. This approach emphasizes long-term, continuous, and dynamic tracking of individuals’ behaviors throughout the entire life cycle. It aims to assess an individual’s status, behavioral trends, and developmental trajectory, empowering users to proactively adjust their lifestyles and promote healthy behaviors [1]. HAR, particularly when applied to daily activity monitoring, plays a central role in this context by helping users understand and manage their physical routines, ultimately facilitating personalized health management.

Among various HAR modalities, wearable sensor-based HAR is especially promising. Compared to vision-based methods [2], it is less intrusive and less affected by environmental variations. Compared to wireless signal-based methods [3], it benefits from a “one device per user” configuration, which avoids inter-user interference and mitigates the need to retrain models in different environments. Furthermore, with the rapid advancement and increasing ubiquity of the Internet of Things (IoT), wearable HAR has received widespread attention. The sensor manufacturing industry has matured significantly, making standardized hardware more affordable and accessible. These trends make wearable sensor-based HAR a low-cost and scalable solution for large-scale deployment.

In recent years, deep learning-based HAR systems have achieved promising results by extracting rich temporal and spatial features from sensor data. However, a key challenge remains: user generalization.

Most existing methods assume that the training and test data are drawn from the same distribution [4,5,6,7,8], which limits their ability to generalize to unseen users.

In real-world applications, sensor signals often vary significantly across individuals due to differences in body shape, movement patterns, sensor placement, and personal habits [9,10]. Consequently, models trained on a fixed user group often fail to generalize to new users—this phenomenon is commonly known as the user generalization problem.

To address this challenge, it is essential to learn user-invariant representations that capture the semantic essence of activities while minimizing user-specific variations. Prior works have explored personalized HAR frameworks [11,12,13,14,15,16], typically adopting a two-stage approach: pre-training on a general dataset followed by fine-tuning on data from the target user. While effective, these methods suffer from two major limitations: (1) collecting data from each new user increases deployment costs, and (2) the two-stage training pipeline adds complexity and computational overhead.

In parallel, several studies have aimed to improve user generalization directly during training by learning domain-invariant representations [10,17,18]. These methods often introduce regularization terms, domain alignment losses, or adversarial objectives to reduce inter-user variability. Although they achieve promising zero-shot performance, such methods usually rely on indirect modeling of user differences and require assumptions such as known domain boundaries or sufficient domain diversity. Moreover, the resulting training pipelines can be complex and sensitive to hyperparameter tuning. In contrast, we take a more direct approach by explicitly leveraging available user labels during training. By formulating a multi-task objective that combines activity classification and supervised contrastive learning based on user and activity labels, our method encourages the model to learn user-invariant yet task-discriminative representations. This strategy avoids domain-specific assumptions and enables efficient generalization to unseen users without requiring per-user adaptation.

In summary, we propose a single-stage learning framework that integrates supervised classification and supervised contrastive learning in a unified multi-task setup. This design enables the model to learn robust and transferable activity representations that generalize well to unseen users, without relying on any user-specific fine-tuning.

The main contributions of this work are summarized as follows:

- We propose a novel multi-task supervised contrastive learning framework for user-generalizable wearable HAR. By jointly leveraging activity and user labels during training, the framework explicitly promotes user-invariant yet activity-discriminative representations, allowing the model to perform user-independent inference without any per-user calibration.
- We introduce a unified single-stage optimization strategy that integrates supervised classification and contrastive objectives into one cohesive learning process. This design avoids the objective misalignment and complexity commonly seen in two-stage pipelines, providing a simple and effective approach for improving user-level generalization.

This paper is organized as follows. Section 2 reviews related work on human activity recognition (HAR) using wearable sensors, outlining recent advances and challenges. Section 3 describes our proposed framework, including its motivation, overall architecture, and key components. Section 4 presents the experimental setup, datasets, and results to evaluate the effectiveness of our approach, including comparisons with baseline methods and ablation studies. Section 5 provides an in-depth analysis of the results and discusses comparisons with related methods and ablation studies. Finally, Section 6 concludes the paper.

## 2. Related Work

### 2.1. Wearable Sensor-Based HAR Model

Sensor-based HAR aims to recognize human activities using various wearable sensors [18]. The wearable sensor-based approach plays an important role in this field due to its advantages in popularity, computational efficiency, and privacy protection, with wearable sensors serving as the main interfaces [19]. Therefore, we focus on the wearable sensor-based approach in this work.

Early practical HAR systems demonstrated the feasibility of recognizing daily activities using body-worn or smartphone sensors in real-world environments. For example, Bao and Intille [20] and Ravi et al. [21] deployed multi-sensor systems to collect data from subjects performing everyday tasks, while Kwapisz et al. [22] validated activity recognition using smartphone accelerometers in unconstrained settings. Subsequent studies such as Weiss et al. [23] further evaluated wearable devices in realistic usage scenarios, providing important insights into device placement and user variability. These works laid the foundation for later HAR research.

In recent years, researchers have proposed various HAR models based on wearable sensors to achieve robust and accurate performance, including feature engineering combined with deep learning models [24] or traditional machine learning models [25], as well as purely deep learning-based approaches [4,5,6,7,8,26,27,28,29].

### 2.2. Contrastive Learning for HAR

Contrastive learning aims to learn discriminative feature representations by contrasting positive and negative sample pairs. Most existing frameworks follow a two-stage pipeline: pre-training with a pretext task and fine-tuning on labeled data. In self-supervised settings, the encoder is trained on unlabeled data and then frozen during fine-tuning, making it especially useful when labeled data are limited.

Owing to this advantage, contrastive learning has been widely adopted in wearable sensor-based human activity recognition (HAR). Haresamudram et al. [30] introduced Contrastive Predictive Coding (CPC) to HAR, leveraging future timestep prediction to encode temporal dependencies. Their self-supervised approach improved performance in low-label regimes and enhanced robustness for transitional activities. Chen et al. [31] proposed SimCLR, which generated two augmented views per sample and trains a Siamese encoder using the NT-Xent loss. Tang et al. [32] applied SimCLR to wearable HAR using a TPN [33] backbone, while Khaertdinov et al. [34] proposed CSSHAR by combining SimCLR with a CNN-Transformer encoder.

A well-known limitation of self-supervised contrastive learning is the presence of false negative pairs. To mitigate this issue, Wang et al. [35] proposed ClusterCLHAR, which employed clustering to reduce false negatives. However, due to the unsupervised nature of clustering, this issue cannot be fully resolved. Alternatively, Prannay et al. [36] introduced Supervised Contrastive Learning (SupCon), where label information was used during pre-training to construct more semantically consistent positive and negative pairs, thereby providing stronger inductive biases.

### 2.3. Personalization and User Generalization Approaches

Despite the success of deep learning in wearable HAR, a persistent challenge remains: the user-dependency problem—models trained on specific users often fail to generalize to unseen individuals due to differences in gait, movement patterns, and sensor placement. Recent studies have explored two main directions to address this issue: personalization and user generalization.

#### 2.3.1. Personalized Approaches

Personalization-based methods adapt models to individual users. CrossHAR [11] learned a shared latent space and applied user-aware recalibration with limited labeled data. Distributed online learning [12] has shown promise for personalized adaptation in streaming IoT scenarios. Saha et al. [13] proposed a lightweight one-dimensional convolutional neural network (CNN), and transfer learning was used to fine-tune the network model using real-time perceived data. On-device training on a smartphone was used for model fine-tuning, enabling the HAR system to achieve personalized customization without compromising privacy or increasing computational costs. Pixi et al. [14] proposed a hybrid framework that combined offline representation learning with on-device classifier adaptation. To address privacy concerns, recent works have explored implicit personalization. IPL-JPDA [15] leveraged pseudo-labeling and multimodal sensing for cross-user adaptation without target labels. Additionally, FedHAR [16] enabled decentralized semi-supervised personalization via prototype-based memory updates and consistency regularization.

While personalized HAR methods effectively handle user heterogeneity, they typically involve two-stage pipelines and require data from target users, raising concerns about privacy and data quality. Although federated and pseudo-labeling approaches offer privacy-preserving alternatives, the lack of reliable target user data remains a key bottleneck for real-world deployment.

#### 2.3.2. User Generalization

Generalization-based methods aim to learn user-invariant representations without relying on target user data. GILE [17] employed variational inference and an independent excitation mechanism to disentangle latent spaces and achieve zero-shot generalization. CCIL [10] was a concept-level regularization strategy that ensured consistency across activity classes by aligning both feature and logit spaces. AFFAR [18] learned domain-invariant and domain-specific features and adaptively fused information from multiple source domains. While effective, these methods often require complex training pipelines or strong assumptions about domain shifts.

While generalization-based methods achieved good zero-shot performance without relying on target user data, their effectiveness often depends on complex regularization objectives and the availability of diverse source domains. These requirements can limit scalability or robustness when applied to real-world scenarios with limited domain variability or resource constraints. In contrast, our approach directly leverages user and activity labels through a multi-task contrastive learning framework, providing a simpler and more interpretable training process while enhancing generalization through explicit semantic alignment. This design offers a viable alternative to complex domain generalization procedures, especially when user labels are available during training.

## 3. Methodology

### 3.1. Problem Setup

The high-level motivation for improving user-level generalization has been introduced in Section 1. Here, we provide the technical rationale and formal problem setup that guide the design of our multi-task supervised contrastive framework.

Given a labeled dataset

$$D={\left\{\left(x_{i},y_{act,i},y_{user,i}\right)\right\}}_{i=1}^{N},$$

where each sample is annotated with both an activity label $y_{act}$ and a user label $y_{user}$, our objective is to learn an encoder $F_{\theta }\left(\cdot \right)$ that produces a representation supporting two properties: (1) accurate activity classification via a downstream classifier $G_{\varphi }\left(\cdot \right)$, and (2) robust generalization to unseen users.

Optimizing the first property is straightforward through supervised learning. However, relying solely on the classification loss does not explicitly enforce user-invariant structure in the representation space. To address this limitation, we incorporate a supervised contrastive learning objective that encourages samples sharing the same activity label to cluster together while pushing apart samples from different activities. Formally, this regularizes $F_{\theta }$ to produce representations that emphasize activity semantics while reducing user-specific variations.

We adopt a multi-task learning (MTL) formulation to jointly optimize the classification and contrastive objectives. MTL enables the encoder to benefit from complementary supervision signals and improves representation robustness across users. Instead of a two-stage pre-training and fine-tuning pipeline, we employ a single-stage joint optimization strategy. This avoids potential objective misalignment between pretext and downstream tasks and simplifies the training process by allowing both objectives to guide representation learning simultaneously.

In summary, the problem setup requires learning activity-discriminative yet user-invariant representations from data annotated with both activity and user labels. Within this formulation, our multi-task supervised contrastive framework provides a unified optimization process in which the classification and contrastive objectives jointly guide the encoder to capture activity semantics while suppressing user-specific variations [37]. This enables the resulting representations to generalize effectively to users unseen during training, without requiring any user-specific adaptation.

### 3.2. Multi-Task Contrastive Learning Framework

**Figure 1:**
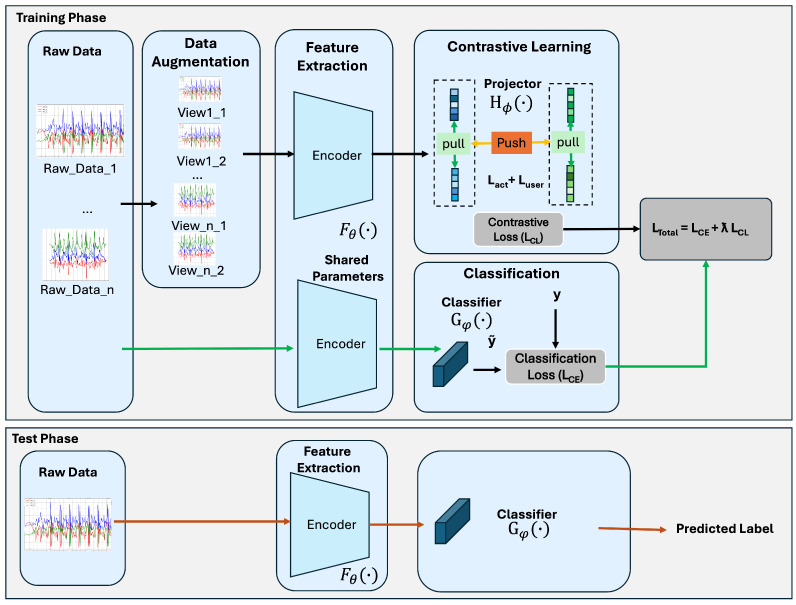
Overview of the proposed Multi-Task Contrastive Learning Framework for User-Generalizable HAR.

An overview of the proposed framework is shown in Figure 1.

As illustrated, during the training phase, data augmentations are applied to generate two distinct views for each input sample. These augmented views are processed by a shared encoder $F_{\theta }\left(\cdot \right)$ to obtain feature representations $f_{\mathrm{cl}}$. In parallel, the raw input data are also passed through the same encoder to produce $f_{\mathrm{raw}}$.

The features $f_{\mathrm{cl}}$ are then fed into a projection head $H_{\phi }\left(\cdot \right)$ to compute the contrastive loss $L_{\mathrm{CL}}$, whereas $f_{\mathrm{raw}}$ is passed to a classification head $G_{\varphi }\left(\cdot \right)$ to compute the classification loss $L_{\mathrm{CE}}$.

#### 3.2.1. Model Architecture

**Figure 2:**
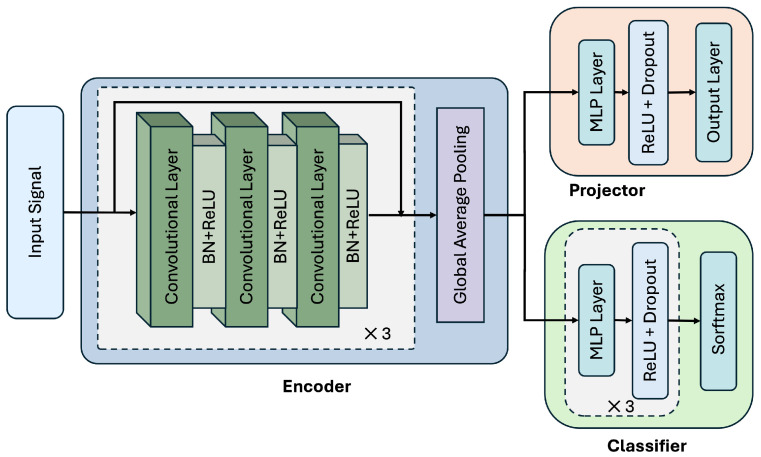
Model architecture. The encoder consists of residual blocks with convolutional layers, followed by global average pooling. The projector and classifier are built from MLP layers with non-linear activations and dropout.

Figure 2 illustrates the overall model architecture used in this work.

To support deployment on wearable devices, where computational resources and battery life are often constrained, we design our framework around a lightweight neural network for efficient feature extraction. In this work, we adopt ResNet [38] as the backbone encoder owing to its strong representational capability and computational efficiency.

As shown in Figure 2, the encoder consists of three stacked residual blocks, each containing three convolutional layers with batch normalization and ReLU activation. A global average pooling layer is applied after the residual blocks to aggregate temporal features into a fixed-length representation. Unless otherwise specified, we follow the original ResNet configuration reported by Wang Z. et al. [38], with only the input and output dimensions adapted to fit each dataset.

It is worth noting that the focus of this work is not on the specific architecture of the encoder. Our framework is model-agnostic and can be flexibly adapted to other backbone networks.

The projection head $H_{\phi }\left(\cdot \right)$ is used exclusively during training for contrastive learning. It projects high-dimensional encoder outputs into a lower-dimensional latent space, serving as an information bottleneck to prevent contrastive signals from directly influencing the encoder. We adopt a non-linear projector composed of one hidden MLP layer with ReLU activation and dropout. The hidden layer size is selected based on the characteristics of each dataset.

The classification head $G_{\varphi }\left(\cdot \right)$ consists of three fully connected MLP layers, each followed by ReLU activation and dropout. The final output layer maps the features to activity logits for classification.

#### 3.2.2. Activity Classification Task

The activity classification task serves as the primary task of our framework. Its goal is to predict the activity label corresponding to a given sensor input. During both training and inference, raw data segments are directly processed by the encoder $F_{\theta }\left(\cdot \right)$ to extract semantic features. These features are then passed to the classification head $G_{\varphi }\left(\cdot \right)$ to produce the predicted class logits.

Let $x_{\mathrm{raw}}$ denote the raw input sample, and let y∈{0,…,C−1} be the corresponding ground-truth activity label, where *C* is the total number of activity classes. The predicted probability distribution over the classes is obtained via the softmax output of the classifier:

(1) $$\hat{y}=\mathrm{softmax}\left(G_{\varphi }\left(F_{\theta }\left(x_{\mathrm{raw}}\right)\right)\right).$$

The classification objective is optimized using the standard cross-entropy loss:

(2) $$L_{\mathrm{CE}}=-\sum_{c=0}^{C-1}y_{c}\cdot \log\left({\hat{y}}_{c}\right),$$

where ${\hat{y}}_{c}$ is the predicted probability of class *c*, and $y_{c}$ is a binary indicator that equals 1 if the true label corresponds to class *c*, and 0 otherwise.

In our multi-task framework, this classification loss $L_{\mathrm{CE}}$ supervises both the encoder $F_{\theta }$ and the classifier $G_{\varphi }$, guiding the model to learn features that are discriminative for activity recognition. During inference, only this classification branch is used to predict activity labels from unseen raw inputs, without relying on augmented views or the projection head.

#### 3.2.3. Contrastive Learning Task

To enhance the generalization ability of the model across different users, we introduce a supervised contrastive learning (SupCon) task as an auxiliary objective during training.

Contrastive learning typically involves four stages: (1) data augmentation, (2) feature extraction, (3) projection and optimization, and (4) fine-tuning with labeled data. In our framework, the contrastive task is incorporated into a multi-task training paradigm, where it shares the encoder with the classification task and is optimized jointly in a single-stage process.

**(a) Data Augmentation.** Each input sample is augmented twice to produce two distinct views, which are then fed into the encoder $F_{\theta }\left(\cdot \right)$ followed by the projection head $H_{\phi }\left(\cdot \right)$, yielding normalized embeddings for contrastive learning.

We adopt a set of simple yet effective augmentations tailored for time-series data [34]. Given a collection of augmentation operators $A=\{a_{1},a_{2},...,a_{n}\}$, each operator is applied to the input signal with a fixed probability *p*. To ensure that every instance is transformed, jittering is applied as a base augmentation (i.e., p = 1). The augmentation methods used include:

- Jittering: Adds Gaussian noise to the signal.
- Scaling: Multiplies the signal by a random scalar drawn from a normal distribution.
- Channel Shuffle: Randomly permutes the channels of multivariate time-series data.
- Rotation: Randomly inverts the sign of the signal values.
- Permutation: Divides the signal into segments and permutes their order.

**(b) Projection Head.** The projection head $H_{\phi }\left(\cdot \right)$ maps the encoder output to a lower-dimensional latent space where the contrastive loss is applied. This component acts as an information bottleneck, ensuring that contrastive supervision does not directly interfere with the encoder’s activity-discriminative space.

To stabilize contrastive learning and enable meaningful similarity comparisons, we apply $\ell_{2}$-normalization to the projected features. Specifically, the output $z_{i}$ of the projection head is normalized as follows:

(3) $$z_{i}=\frac{H_{\phi }\left(f_{i}\right)}{∥H_{\phi }\left(f_{i}\right){∥}_{2}},$$

where $f_{i}$ denotes the encoder output for the *i*-th input sample, and ${∥\cdot ∥}_{2}$ represents the Euclidean norm. This operation projects all feature vectors onto the unit hypersphere, ensuring unit length.

Such normalization is essential in contrastive learning frameworks such as SimCLR [32] and SupCon [36], where similarity is computed via the dot product (cosine similarity). Without normalization, the model may exploit feature magnitudes rather than directions to minimize the loss, leading to unstable training or representation collapse. Moreover, this step enhances the robustness of the learned representations, particularly under small batch sizes or high-temperature settings in the contrastive loss.

**(c) Positive and Negative Sampling.** The construction of positive and negative sample pairs is crucial for the effectiveness of contrastive learning. We adopt a hard negative sampling strategy tailored to the user generalization challenge. Specifically, given the projected embedding $z_{i}$ of the *i*-th view and its corresponding activity label $y_{i}$, we define:

- Positive pairs: samples with the same activity label across different users.
- Negative pairs: samples with different activity labels but from the same user.

This design encourages the model to learn activity-discriminative yet user-invariant features by forcing it to ignore user-specific variations that are not semantically meaningful.

**Figure 3:**
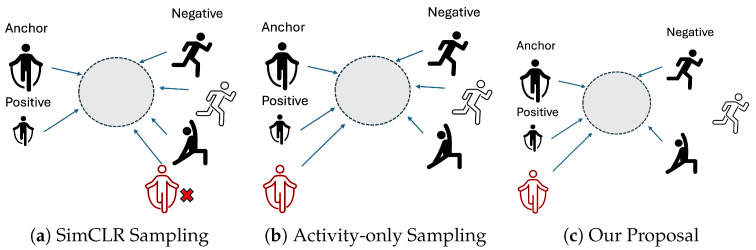
Comparison of positive and negative pair construction strategies.

Figure 3 illustrates how our method differs from traditional SimCLR or activity-only supervised contrastive learning in the construction of positive and negative pairs. By mining hard negatives from the same user but different activities, our approach encourages the model to suppress subject-specific biases and learn user-invariant features more effectively.

**(d) Contrastive Loss.** We adopt the supervised contrastive loss (SupCon) [36], defined as:

(4) $$L_{\mathrm{CL}}=\sum_{i\in I}\frac{-1}{\left|P(i)\right|}\sum_{p\in P(i)}\log\frac{\exp(z_{i}\cdot z_{p}/\tau )}{\sum_{a\in A(i)}\exp\left(z_{i}\cdot z_{a}/\tau \right)},$$

where τ is a temperature hyperparameter, I is the index set of all anchors in the batch, P(i) is the set of positives for anchor *i*, and A(i) includes all positives and negatives except *i* itself. The similarity is measured using the dot product between the projected embeddings.

**(e) Training and Inference.** The contrastive loss is applied only during training and backpropagates through the encoder and projection head. It is discarded during inference. Nevertheless, it serves as a strong regularizer that improves the robustness and transferability of the learned features, thereby enhancing user-level generalization in the classification task.

#### 3.2.4. Loss and Optimization

The total loss is defined as a weighted combination of the two objectives:

(5) $$L_{\mathrm{total}}=L_{\mathrm{CE}}+\lambda \cdot L_{\mathrm{CL}},$$

where λ∈[0,1] controls the trade-off between the main and auxiliary tasks.

During optimization, the model parameters are updated as follows:

(6) $$\left\{\begin{array}{cc} \theta & \leftarrow \theta -\alpha \left(\frac{\partial L_{\mathrm{CE}}}{\partial \theta }+\lambda \frac{\partial L_{\mathrm{CL}}}{\partial \theta }\right),\\ \varphi & \leftarrow \varphi -\alpha \left(\frac{\partial L_{\mathrm{CE}}}{\partial \varphi }\right), \end{array}\right.$$

where α denotes the learning rate. In our training scheme, the classification loss $L_{\mathrm{CE}}$ updates both the encoder $F_{\theta }\left(\cdot \right)$ and the classifier $G_{\varphi }\left(\cdot \right)$, while the contrastive loss $L_{\mathrm{CL}}$ updates only the encoder.

During testing, the raw data are directly fed into the encoder to extract features, which are then passed through the classifier to obtain the predicted activity labels.

### 3.3. Evaluation Metrics

We adopt the macro-averaged F1 score as the primary evaluation metric. It is defined as:

(7) $$F1=\frac{2}{C}\sum_{c=0}^{C-1}\frac{p_{c}\cdot r_{c}}{p_{c}+r_{c}},$$

where *C* denotes the total number of activity classes, and $p_{c}$ and $r_{c}$ represent the precision and recall for class *c*, respectively.

Macro-averaging treats all classes equally by computing the unweighted mean across classes, which makes it more robust to class imbalance—a common issue in human activity recognition datasets.

## 4. Experiments

### 4.1. Dataset and Preprocessing

We evaluated our framework on three public datasets that are widely used as benchmarks in this field. These datasets include diverse participants, activities, and sensor configurations, providing standardized benchmarks for evaluating user-level generalization and ensuring fair comparison across studies. Table 1 summarizes the basic information of the dataset.

#### 4.1.1. MobiAct [39]

The MobiAct dataset, published in 2016, was collected using a smartphone. It includes data from 66 participants aged between 20 and 47 years, who performed 16 types of activities: 4 types of falls and 12 types of Activities of Daily Living (ADLs). The recorded signals include acceleration (x, y, z axes), angular velocity (x, y, z axes), and orientation (azimuth, pitch, roll). The acceleration data range is ±2 g.

The dataset contains a total of 3199 samples, comprising 767 falls and 2432 ADLs, sampled at 200 Hz. In this study, data from 11 types of ADLs are used for experiments: standing (STD), walking (WAL), jogging (JOG), jumping (JUM), going upstairs (STU), going downstairs (STN), sitting (SIT), standing up from a chair (CHU), sitting down on a chair (SCH), stepping into a car (CSI), and stepping out of a car (CSO). The lying data are excluded because they were recorded as part of the falling process and therefore do not represent independent ADL activities.

#### 4.1.2. UCI HAR [40]

The UCI HAR dataset, published in 2013, contains data collected from 30 participants aged between 19 and 48 years. The data were recorded using a smartphone worn on the waist. This dataset focuses on six types of ADLs: walking, walking upstairs, walking downstairs, sitting, standing, and lying.

The dataset comprises 10,299 samples, each lasting 2.56 s and sampled at 50 Hz.

#### 4.1.3. USC-HAD [41]

The USC-HAD dataset, published in 2012, contains data collected from 14 participants aged between 21 and 49 years. The data were recorded using an Inertial Measurement Unit (IMU) worn on the right hip. This dataset includes 12 types of ADLs: walking forward, walking left, walking right, walking upstairs, walking downstairs, running forward, jumping, sitting, standing, sleeping, taking the elevator up, and taking the elevator down.

The dataset comprises 840 samples, each sampled at 100 Hz.

To facilitate comparison and data processing, we downsampled all datasets to 50 Hz in the experiments and divided the data into 1 s windows with a 50% overlap. We downsampled the datasets to 50 Hz for two main reasons: (1) Standardization and comparability: A 50 Hz sampling rate is a common and well-established practice in the HAR literature. This standardization allows for consistent window sizes in terms of time duration. (2) Signal characteristics: According to the Nyquist theorem, a 50 Hz sampling rate is sufficient to capture all signal dynamics up to 25 Hz. For human-scale activities (e.g., walking, running, jumping), most discriminative information lies in low-frequency bands, typically well below 10–15 Hz [42]. Downsampling to 50 Hz can reduce computational amount without losing the core kinematic patterns required for accurate activity classification. Unless otherwise stated, datasets are split by users. For each dataset, we allocated 20% of users’ data as the validation set and 20% as the test set, with the remaining 60% used for training.

### 4.2. Implementation Details

In this work, we use the AdamW [43] optimizer. For each dataset, the training hyperparameters were fine-tuned, as detailed in Table 2. All experiments were implemented in PyTorch 2.7.1 on a local workstation equipped with an Apple M3 Pro GPU (Apple Inc., Cupertino, CA, USA) running macOS Sonoma 14.3. The backbone configuration follows the original ResNet configuration reported by Wang, Z et al. [38] unless otherwise specified.

### 4.3. Main Results

To comprehensively evaluate the effectiveness of our proposed method, we compare it against a wide range of baseline approaches spanning multiple learning paradigms:

- Supervised Baselines: Traditional HAR models trained in a fully supervised manner, leveraging various backbone architectures such as the hybrid CNN-Transformer-BiLSTM (CTBL) [27], and the convolutional autoencoder (CAE) [28], DeepConvLSTM [29], CNN-Transformer (Sup. CSSHAR) [34].
- Self-Supervised Learning Baselines: Contrastive or predictive representation learning approaches trained in two stages. This group includes Contrastive Predictive Coding (CPC) [30], CSSHAR [34], and ClusterCL-HAR [35].
- Personalized Baselines: Approaches tailored for personalized HAR through user-specific fine-tuning or model adaptation. Representative methods include ProtoHAR [44] and FedHAR [16].
- User-Generalization Baselines: Methods explicitly designed to enhance user-level generalization and mitigate subject-domain shifts in wearable sensor-based HAR. This category includes GILE [17], CCIL [10], AFFAR [18], and Multi-task SSL [33].

Together, these baselines cover a diverse spectrum of learning strategies—from traditional supervised models to advanced self-supervised frameworks—and provide a solid foundation for evaluating the generalization performance of our method across users and datasets.

We performed three independent user-disjoint validation runs. The results are summarized in Table 3.

Table 3 presents the macro-F1 scores of our proposed method and a range of baseline methods on three benchmark HAR datasets: MobiAct, UCI-HAR, and USC-HAD. As shown, our method MultiSupConHAR achieves the best performance on MobiAct (85.93% ± 1.23%) and USC-HAD (76.84% ± 1.09%), and ranks second on UCI-HAR (91.07% ± 2.09%), slightly behind supervised CSSHAR (93.73% ± 1.02%), which employs a CNN-Transformer backbone, and CTBL (92.72% ± 1.48%), which employs a CNN-Transformer-BiLSTM backbone.

Compared with standard supervised baselines such as DeepConvLSTM and CTBL, our method shows significant improvements, especially on the USC-HAD dataset, with gaining ranging from 9.99% to 16.57% improvement. On USC-HAD, our method outperforms all other methods, including personalized and generalized HAR approaches, demonstrating strong generalization capability across subjects.

Moreover, MultiHAR also achieves consistently better performance than contrastive self-supervised methods such as CSSHAR, Multi-task SSL, CPC, CAE, and ClusterCLHAR, indicating the benefit of combining contrastive learning with supervised classification in a multi-task framework.

We also report the model size, FLOPs, and memory usage in Table 4. Table 5, Table 6 and Table 7 present the test classification reports of our method on the three datasets.

### 4.4. Ablation Study

To gain deeper insights into the effectiveness of the proposed multi-task contrastive learning framework, we conduct a series of ablation studies under the LOSO (Leave-One-Subject-Out) evaluation setting. These experiments are designed to disentangle the contributions of individual design components and provide empirical justification for each design choice.

Specifically, we examine the following aspects:

- Effectiveness of Multi-Task Training: We compare three settings—supervised classification only (primary task), supervised contrastive learning followed by downstream classification (auxiliary task only), and our joint multi-task training approach.
- Contrastive Learning Strategies: We investigate different strategies for constructing positive and negative pairs, including with/without user labels, with/without activity labels, and compare two-stage versus single-stage training schemes.
- Auxiliary Task Weight(λ): We vary the weight of the contrastive loss in the total loss function, testing λ∈{0.1,…,0.9} to observe its influence on model performance.
- Hyperparameter Sensitivity: We study the impact of key hyperparameters, including batch size, the presence of a projection head, and the hidden dimensionality of the projection head.

These ablation analyses aim to address the following research questions:

1. Does joint multi-task training yield better HAR classification performance than training on individual tasks alone?
2. How should positive and negative pairs be constructed? Is incorporating user identity during training beneficial?
3. Is the proposed single-stage multi-task approach more effective than a two-stage contrastive pre-training followed by fine-tuning?
4. How sensitive is model performance to the choice of contrastive loss weight (λ)?
5. Are the selected hyperparameters (e.g., batch size, projection head) optimal for both performance and generalization?

All experiments in this section use the same ResNet backbone.

#### 4.4.1. Effectiveness of Multi-Task Training

To evaluate the effectiveness of combining classification and contrastive learning in a multi-task framework, we compare the following variants:

- Supervised Classification Only (Primary Task Only): The model is trained solely with the cross-entropy loss for activity classification.
- SupCon Only (Act + User): A two-stage training approach in which the model is first trained using the supervised contrastive loss with both activity and user labels. The encoder is then frozen, and a classifier is fine-tuned on the fully labeled dataset.
- MultiSupConHAR (SupCon Act + User): Our proposed method, where the model is trained end-to-end by jointly optimizing the classification loss and the supervised contrastive loss.

This comparison allows us to evaluate whether multi-task training provides a synergistic advantage over single-objective approaches.

The results are summarized in Table 8.

Table 8 presents the macro-averaged F1 scores of different training paradigms on the three HAR datasets. Our proposed multi-task framework consistently outperforms both the supervised-only model and the two-stage SupCon model across all datasets.

Specifically, the proposed method achieves 86.01% on MobiAct, 93.16% on UCI-HAR, and 77.13% on USC-HAD. Compared with the supervised-only baseline, the performance improvements are 10.88%, 3.45%, and 5.78%, respectively. When compared with the SupCon-only variant, our method also yields consistent gains of 3.20%, 0.94%, and 9.99% on the three datasets.

These results demonstrate that jointly optimizing the classification and contrastive objectives within a unified training framework leads to superior performance compared with using either objective alone.

#### 4.4.2. Contrastive Strategy Analysis

To examine the impact of different contrastive learning strategies on user-level generalization, we compare several variants based on how positive and negative pairs are constructed, and whether contrastive learning is applied in a two-stage or multi-task manner. Specifically, we consider the following settings:

- SimCLR (Two-stage): Self-supervised contrastive learning based solely on data augmentations, without using any labels. The construction of positive and negative pairs follows Figure 3a. The encoder is then frozen, and a classifier is fine-tuned on the fully labeled dataset. The contrastive loss is computed using the XNent loss Formulation (8) [34].
- SupCon (Act Only, Two-stage): Supervised contrastive learning using only activity labels to construct positive and negative pairs (Figure 3b). The encoder is then frozen, and a classifier is fine-tuned on the fully labeled dataset.
- SupCon (Act + User, Two-stage): A stricter version of SupCon, in which both activity and user labels must match to form positive and negative pairs (Figure 3c).
- Multi-task + SimCLR: A joint training approach where SimCLR-based self-supervised contrastive learning is used as an auxiliary task alongside activity classification (Figure 3a). The contrastive loss is computed using the XNent loss Formulation (8) [34].
- Multi-task + SupCon (Act Only): Joint training with SupCon using activity labels (Figure 3c).

(8) $$L_{\mathrm{XNent}}=\sum_{i=1}^{N}-\log\frac{\exp(z_{i}\cdot z_{p}/\tau )}{\sum_{j\in A(i)}\exp\left(z_{i}\cdot z_{j}/\tau \right)}$$

This comparison aims to address the following questions:

- Does leveraging label information in contrastive learning improve downstream HAR performance?
- Does incorporating both user and activity identities into positive sampling help the model learn more user-invariant features?
- Do multi-task learning variants outperform their two-stage counterparts across different strategies?

For the self-supervised contrastive learning settings, we followed dataset-specific hyperparameter configurations as reported in previous studies [34,45]. Specifically, during the pre-training stage, we used a learning rate of 0.0001 for MobiAct and UCI-HAR, and 0.00001 for USC-HAD. During the fine-tuning stage, a fixed learning rate of 0.0001 was used for all datasets. All other hyperparameters were kept consistent with those used in the MultiSupConHAR setting.

In the two-stage training pipeline, the encoder was frozen during the fine-tuning stage, and only the classifier was trained. All self-supervised models were pre-trained for 200 epochs and subsequently fine-tuned for another 100 epochs.

The results are presented in Table 9.

As shown in Table 9, our proposed method outperforms all contrastive learning strategies across the three datasets.

Among the baselines, supervised contrastive learning using activity labels (SupCon Act Only) performs better than the self-supervised SimCLR approach in both two-stage and multi-task settings. The multi-task variants (SimCLR or SupCon) consistently outperform their corresponding two-stage counterparts. Furthermore, the combination of activity and user label supervision (Act + User) under multi-task training yields the best overall results.

#### 4.4.3. Auxiliary Task Weight Analysis

**Figure 4:**
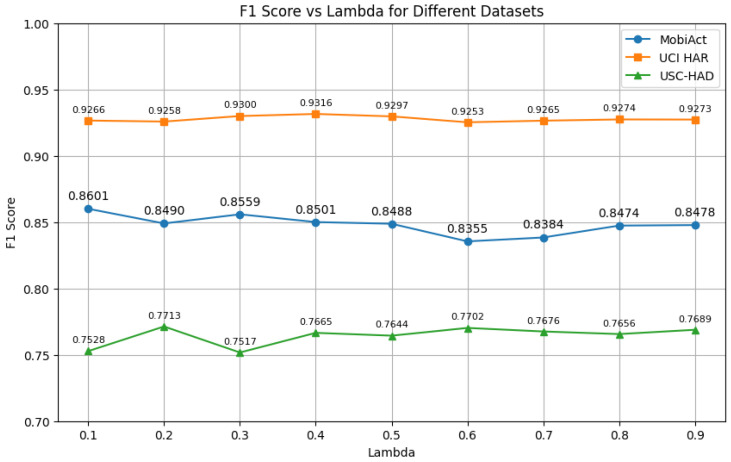
Effect of contrastive loss weight λ on model performance (F1 score) across different datasets.

We further investigate the impact of the loss balancing weight λ, which controls the contribution of the contrastive objective within the multi-task training framework. We vary λ from 0.1 to 0.9 and report the macro-F1 scores on the three datasets, as shown in Figure 4.

Overall, we observe that incorporating the contrastive loss (λ > 0) consistently improves performance compared with purely supervised learning across all datasets. For MobiAct, the best performance is achieved at λ = 0.1, while USC-HAD performs best at λ = 0.2. In contrast, UCI-HAR exhibits relatively stable performance across a wider range of λ values, with the highest score observed at λ = 0.4.

These results suggest that the optimal value of λ may vary depending on dataset characteristics. Datasets with higher inter-class similarity or more diverse activity types (such as MobiAct and USC-HAD) appear to be more sensitive to the choice of λ, requiring a careful balance between the main classification and auxiliary contrastive objectives. In general, setting λ to a value below 0.5 yields better performance, indicating that a moderate contrastive loss weight is sufficient to enhance representation learning without overwhelming the primary classification task.

Interestingly, performance degrades slightly when λ is too large (e.g., 0.6–0.7 on MobiAct), possibly due to the overemphasis on the auxiliary task. These findings indicate that careful tuning of λ is beneficial, and that incorporating contrastive learning as an auxiliary task with a moderate weight enhances cross-user generalization without compromising classification performance.

#### 4.4.4. Hyperparameter Analysis

We further investigate the sensitivity of our model to several key hyperparameters within the contrastive learning framework. Specifically, we evaluate the effects of batch size, the presence or absence of a projection head, and the hidden dimensionality of the projection head. These factors are known to influence the learning dynamics and the quality of the learned representations in contrastive learning [46]. All experiments were conducted under the multi-task training setup with SupCon (Act + User) on the three datasets, while keeping all other settings fixed.

**Figure 5:**
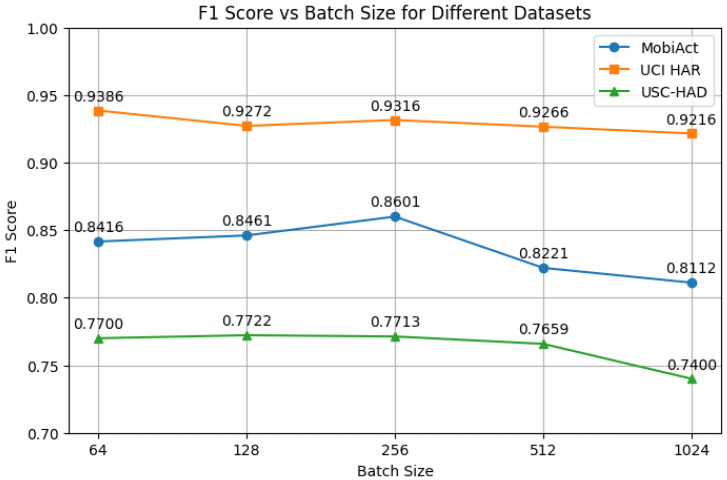
Effect of batch size on F1 score across three datasets.

**(a) Batch Size.** We evaluated the effect of batch size on model performance by varying it across {64, 128, 256, 512, 1024}. The results, shown in Figure 5, indicate that moderate batch sizes generally lead to better performance across all datasets.

On the MobiAct dataset, performance peaks at a batch size of 256 with a macro-F1 score of 86.01%, while larger batch sizes (e.g., 512 and 1024) result in noticeable degradation. A similar trend is observed on USC-HAD, where performance decreases significantly from 77.13% at a batch size of 256 to 74.00% at a batch size of 1024. In contrast, UCI-HAR appears relatively less sensitive to the batch size, although a slight decline in performance is observed as the batch size increases.

**Figure 6:**
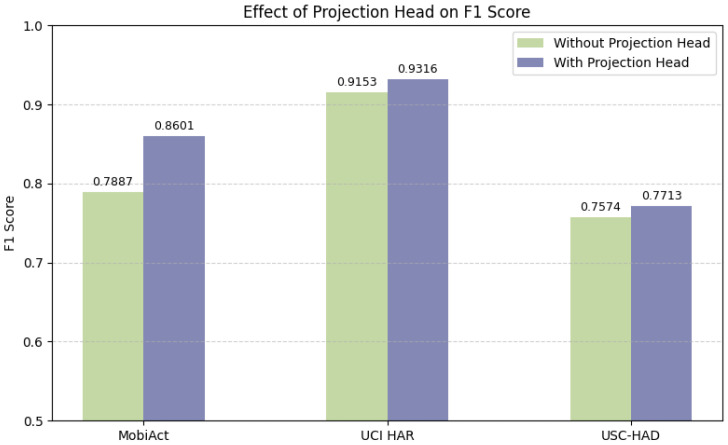
Effect of projection head on F1 score across three datasets.

**(b) Projection Head.** To assess the impact of the projection head in the contrastive learning task, we compare model performance with and without a two-layer MLP projection head placed before the contrastive loss. As shown in Figure 6, incorporating a projection head consistently improves the F1 score across all datasets. The most significant improvement is observed on the MobiAct dataset, where the F1 score increases from 78.87% to 86.01%. Smaller but consistent gains are also observed on UCI-HAR and USC-HAD.

**Figure 7:**
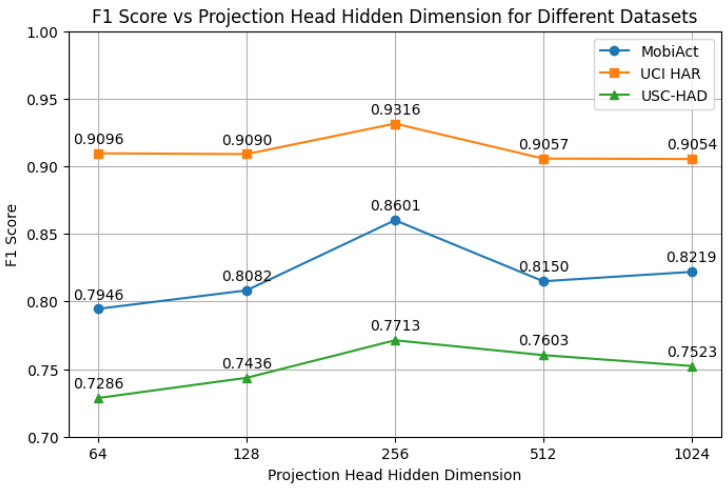
Effect of hidden dimension size in the projection head on F1 score.

We further examine the effect of the hidden dimension size in the projection head. As shown in Figure 7, we evaluate hidden dimensions of {64, 128, 256, 512, 1024}. The results indicate that a moderate hidden size (256) yields the best performance across all datasets.

## 5. Discussion

As demonstrated in the previous section, our method—MultiSupConHAR—achieved performance comparable to the baselines, demonstrating its effectiveness in enhancing user generalization in HAR.

### 5.1. Main Results and Comparisons

Our proposed method outperforms most baseline models across the three HAR datasets and achieves the best overall performance. Although its performance is slightly lower than that of Sup. CSSHAR and CTBL on the UCI-HAR dataset, the results demonstrate that introducing supervised contrastive learning effectively enhances user generalization. Furthermore, as shown in Table 4, although our method has a slightly larger number of parameters compared to the classic DeepConvLSTM baseline, it has lower Flops and memory usage during inference because it does not use recurrent structures such as LSTM.

Compared with self-supervised contrastive learning methods, our approach not only achieves higher accuracy but also eliminates the need for a two-stage training process. This design reduces training costs and mitigates the potential risk of objective mismatch between the pre-training and fine-tuning stages. For instance, CSSHAR [34] adoptd a SimCLR-based framework with a CNN–Transformer encoder. Its model complexity is relatively high (9.3 M, 5.4 M, and 6.6 M parameters on the three datasets), whereas our method uses a ResNet encoder with only 481K parameters, resulting in significantly lower computational and energy demands—making it more suitable for edge-device deployment. Other methods, such as CPC [30] and ClusterCLHAR [35], also involved two-stage training. CPC (Contrastive Predictive Coding) learned temporal dependencies by predicting future latent representations from past context vectors. ClusterCLHAR performed clustering-based instance discrimination during pre-training. Both methods were designed to leverage unlabeled data by pre-training on unannotated signals and subsequently fine-tuning on labeled data. While this strategy helps alleviate label scarcity, it does not improve cross-user generalization under fully supervised settings.

We also compared our method with personalization-based approaches such as ProtoHAR [44] and FedHAR [16], which employed federated learning to build user-specific models. Both methods first trained a general model and then fine-tuned personalized models on client devices. This design effectively addresses privacy concerns by keeping user data local. However, they still require new user data to be collected and labeled, followed by local re-training—again, a two-stage process. In contrast, our method achieves better performance within a single-stage pipeline without requiring per-user re-training.

Existing approaches to user generalization in HAR can be broadly categorized into three directions:

1. Self-supervised pretraining (e.g., Multi-task SSL [33]) learns transformation-aware representations through auxiliary tasks. While effective for representation learning, these methods typically rely on separate pretraining and fine-tuning stages, which limits task-level integration.
2. Domain disentanglement methods (e.g., GILE [17]) aim to separate domain-invariant and domain-specific features through probabilistic modeling. These approaches enable zero-shot transfer but involve complex, sampling-based training procedures.
3. Alignment-based strategies(e.g., CCIL [10], AFFHAR [18]) introduce explicit mechanisms (e.g., concept matrices, domain alignment losses) to enforce consistency across users or domains. Although these methods encourage generalization, they add extra training components and regularization overhead.

Our method offers an alternative perspective by unifying classification and supervised contrastive learning within a multi-task setting. This formulation leverages both activity and user labels to directly guide representation learning, without relying on external alignment modules or multi-stage training.

This design enables end-to-end optimization and promotes stable training dynamics. Moreover, the encoder remains compact (only 481K parameters), making it well-suited for deployment on resource-constrained devices. As demonstrated by our experiments, the proposed approach achieves competitive user-level generalization while maintaining a simple and modular architecture.

Overall, the framework provides a balanced integration of task-driven supervision and contrastive learning, offering a practical pathway toward generalizable HAR under real-world constraints.

### 5.2. Ablation Study Discussion

Our proposed method is built upon two key design choices. First, we introduce contrastive learning as an auxiliary task and jointly optimize it with the primary classification task in an end-to-end manner. Second, we adopt a supervised contrastive loss that utilizes both activity and user labels to construct positive and negative pairs, enabling user-aware representation learning.

As shown in Table 8, the multi-task setting outperforms individual single-task settings, validating the effectiveness of our joint optimization strategy.

As shown in Table 9, our proposed multi-task method consistently outperforms all contrastive learning variants across the three datasets. We highlight several key observations:

1. Supervised contrastive learning (SupCon) achieves higher performance than self-supervised contrastive learning (SimCLR), indicating that label supervision is beneficial for wearable HAR tasks.
2. Multi-task variants consistently outperform their two-stage counterparts, highlighting the advantages of end-to-end joint training in balancing generalization and optimization stability.
3. Interestingly, while incorporating both activity and user labels (Act + User) into the contrastive learning process improves performance in the multi-task setting, we observe limited or no improvement in the two-stage setting. This difference may arise from how the two paradigms utilize supervision signals during optimization.

In the two-stage setting, the encoder is pre-trained solely using the contrastive loss, independent of the downstream classification objective. Although user labels are used to guide the sampling of positive and negative pairs, the learned representations are not explicitly aligned with the classification task. As a result, the semantic structure induced by user supervision may not transfer effectively to the downstream task and may even interfere with fine-tuning due to objective misalignment.

By contrast, the multi-task setting jointly optimizes both contrastive and classification losses. This end-to-end formulation ensures that user-level supervision is integrated in a way that remains compatible with the classification objective. The contrastive loss shapes the feature space by introducing user-level discrimination, while the classification loss anchors the representations around activity semantics. We hypothesize that this synergy improves hard negative mining and leads to more transferable representations across users.

Moreover, joint training reduces the risk of representation drift between the pre-training and fine-tuning stages—a common issue in two-stage pipelines where the learned contrastive space is decoupled from the final task objective.

These findings support our design choice: integrating activity- and user-aware contrastive learning into a multi-task framework offers a favorable trade-off between user generalization and learning stability.

Figure 4 illustrates the impact of the contrastive loss weight λ on model performance.

The optimal value of λ varies across datasets. Datasets with higher inter-class similarity or a larger number of activities (e.g., MobiAct and USC-HAD) appear more sensitive to λ, requiring careful calibration to balance the main classification and auxiliary contrastive objectives.

Overall, setting λ < 0.5 yields better performance, suggesting that a moderate contrastive loss weight is sufficient to enhance feature learning without overwhelming the primary classification task. In contrast, performance slightly degrades when λ is too large (e.g., 0.6–0.7 on MobiAct), possibly due to an overemphasis on the auxiliary objective. These results confirm the importance of tuning λ to achieve robust and generalizable performance.

We further investigate the effects of batch size, the presence of a projection head, and the hidden dimensionality of the projection head. The corresponding results are shown in Figure 5, Figure 6 and Figure 7.

- Batch Size: Consistent with prior studies [46], excessively large batch sizes can reduce gradient diversity and introduce optimization instability. We select a batch size of 256 to balance computational efficiency and model performance.
- Projection Head: The inclusion of a projection head improves performance, aligning with previous findings in contrastive learning [32]. The projection head serves as a representation bottleneck, decoupling the contrastive space from the classification space and thereby enhancing generalization.
- Hidden Dimension: Using overly small (e.g., 64) or large (e.g., 1024) hidden dimensions leads to performance degradation. This suggests that under-parameterization limits representational capacity, while over-parameterization may cause overfitting or training instability. A moderate hidden dimension (e.g., 256) provides the best trade-off.

These ablation results comprehensively validate each component of our framework and highlight the robustness and generalizability of our multi-task contrastive learning approach for user-level HAR.

**Figure 8:**
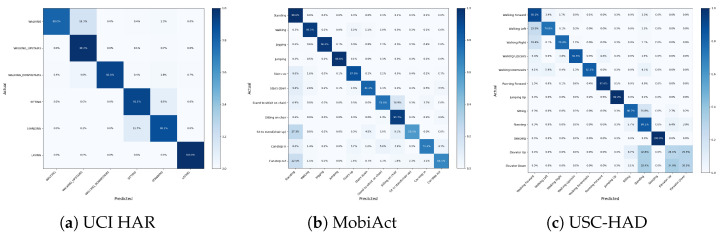
Comfusion Matrix.

Figure 8 shows the confusion matrices of our model on the UCI HAR, MobiAct, and USC-HAD datasets. Overall, the model achieves a high diagonal advantage on all datasets, indicating that most activities are correctly identified. However, some systematic misclassifications are still observed.

For the UCI HAR dataset, the main confusion occurs between “walking” and “walking upstairs,” and between “sitting” and “standing.” These activities have similar dynamic patterns or transitional postures, leading to overlapping temporal features in the sensor space.

In the MobiAct dataset, although there are many activity categories, most categories are accurately distinguished. However, transitional actions such as “standing to sitting/sitting in a chair” and “sitting to standing (chair raised)” exhibit significant confusion (e.g., mutual misclassification rates of approximately 15–25%), reflecting the challenge of identifying short-term transitional states with subtle kinematic differences. Significant confusion also occurs between “standing” and “car step-in/car step-out.” Short-term transitional states also exist between these actions.

For the USC-HAD model, errors primarily occur in walking-related activities (e.g., walking forward, left, and right), due to the similar periodic motion patterns of these activities. Furthermore, elevator movements (going up and down) are frequently confused with standing, likely because these movements involve smaller amplitude of body motion or because data such as acceleration and angular velocity during the uniform motion phase of an elevator ride are consistent with the standing state.

These observations suggest that while the proposed model captures discriminative representations of most activities, further improvement may require temporal context modeling or explicit transition state enhancement to better distinguish between fine and low-motor activities.

**Figure 9:**
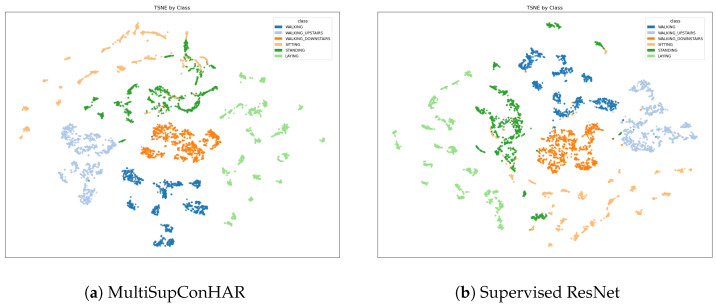
t-SNE visualization of representations (UCI HAR).

**Figure 10:**
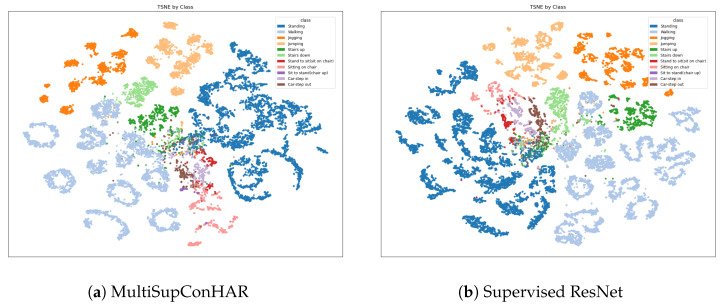
t-SNE visualization of representations (MobiAct).

**Figure 11:**
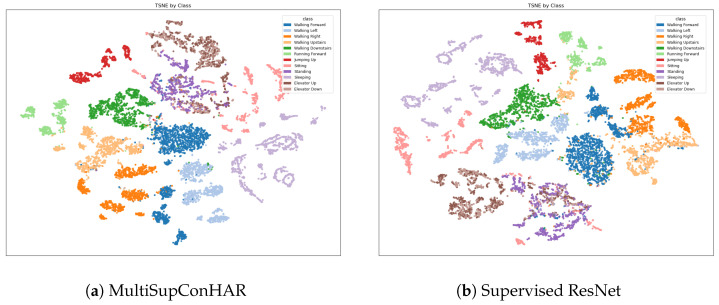
t-SNE visualization of representations (USC-HAD).

Figure 9, Figure 10 and Figure 11 shows the t-SNE visualization of representations on the UCI HAR, MobiAct, and USC-HAD datasets of our proposal and supervised ResNet.

## 6. Conclusions and Future Work

This study presented a multi-task contrastive learning framework for user-generalizable human activity recognition (HAR) using wearable sensor data. By jointly optimizing supervised classification and contrastive objectives with both activity and user labels, the proposed method effectively enhances feature representations without requiring user-specific adaptation. Extensive cross-user evaluations on three public datasets verified its effectiveness and robustness, outperforming baseline and state-of-the-art methods on MobiAct and USC-HAD, and achieving comparable results on UCI HAR. The ablation analyses further confirmed the benefits of joint training, supervised contrastive loss, and task-aware pair construction.

At the same time, the findings of this work highlight several directions for further research.

First, since the framework relies on fully labeled data for both activity and user identities, future studies could explore semi-supervised and self-supervised extensions—such as pseudo-labeling, contrastive pretraining, or weak supervision—to reduce annotation requirements.

Second, validation was limited to public datasets. Additional in-the-wild deployment and long-term evaluation would provide stronger evidence of robustness to variations in sensor placement and user lifestyle.

Finally, the present framework mainly targets cross-user generalization; integrating complementary strategies such as domain adaptation, meta-learning, or adversarial data generation may enable broader cross-domain and cross-device transferability.

Moreover, insights from recent computer vision research—such as multiview attention networks with random interpolation–based augmentation [47], content–style contrastive frameworks for domain generalization [48], and weakly supervised adversarial segmentation approaches [49] could inspire future extensions of sensor-based HAR towards more robust and adaptive models.

Overall, the proposed framework provides methodological contributions and outlines a potential direction for developing scalable and label-efficient user-generalizable HAR systems.

## Figures and Tables

**Table 1:** Datasets.

Dataset	Classes	Frequency	Sensors	Subject
MobiAct [39]	11	200 Hz	A, G, O	66
UCI HAR [40]	6	50 Hz	A, G	30
USC-HAD [41]	12	100 Hz	A, G	14

A: Accelerometer; G: Gyroscope; O: Orientation (ignored).

**Table 2:** Training settings.

Dataset	α	Projection Size	Batch Size	τ	Epochs (*ES*)	λ
MobiAct	0.0003	256	256	0.1	200 (30)	0.2
UCI HAR	0.0003	256	256	0.1	100 (30)	0.4
USC-HAD	0.0001	256	256	0.1	200 (30)	0.3

α: learning rate. τ: temperature. *ES*: Early Stopping Patience. λ: Auxiliary weight.

**Table 3:** Macro-F1 Score (%) on Three HAR Datasets (Ave. ± STD.).

Type	Method	MobiAct	UCI-HAR	USC-HAD
Sup.	DeepConvLSTM [29]	82.40 ± 1.82	82.64 ± 0.86	67.14 ± 2.56
	Sup. CSSHAR [34]	82.97 ± 1.10	**93.73** ± **1.02**	59.53 ± 1.06
	CTBL [27]	78.66 ± 5.30	92.72 ± 1.48	69.11 ± 4.29
	CAE [28]	78.75 ± 1.76	79.82 ± 0.97	49.88 ± 1.87
SSL	CSSHAR [34]	80.22 ± 1.02	90.51 ± 0.60	60.57 ± 1.92
	CPC [30]	81.54 ± 1.30	82.08 ± 1.04	52.31 ± 1.95
	ClusterCLHAR ^*^ [35]	-	92.12	58.85
Pers.	ProtoHAR ^*^ [44]	-	-	71.71
	FedHAR ^*^ [16]	-	79.34	-
Gen.	Multi-task SSL [33]	76.40 ± 1.59	82.30 ± 1.36	49.83 ± 3.58
	GILE ^*^ [17]	-	88.17	-
	CCIL ^*^ [10]	-	-	57.5
	AFFAR ^*^ [18]	-	-	72.58
**Ours**	**MultiSupConHAR**	**85.93 ± 1.23**	91.07 ± 2.09	**76.84 ± 1.09**

^*^: Official results reported in original paper. Bold numbers indicate the best performance on each dataset.

**Table 4:** Model Size, FLOPs, and Memory.

Method	Metric	MobiAct	UCI HAR	USC-HAD
DeepConvLSTM	Model size	458.00 k	458.00 k	458.00 k
	FLOPs (Inference)	53.20 M	53.20 M	53.20 M
	Memory (Inference)	2.35 M	2.35 M	2.35 M
CSSHAR	Model size (parameters)	9.30 M	5.40 M	6.60 M
	FLOPs (Inference)	823.70 M	491.44 M	614.75 M
	Memory (Inference)	48.40 M	26.98 M	31.59 M
MultiSupConHAR	Model size (parameters)	565.60 k	566.00 k	566.40 k
	FLOPs (Inference)	48.50 M	48.50 M	48.50 M
	Memory (Inference)	2.20 MB	2.20 MB	2.20 MB

**Table 5:** Classification Report on UCI HAR (%).

Class	Precision	Recall	F1 Score	Support
Walking	99.48	80.00	88.68	950
Walking upstairs	81.28	99.16	89.33	950
Walking Downstairs	100.0	92.02	95.85	890
Sitting	86.65	91.06	88.80	962
Standing	88.67	88.09	88.38	1075
Laying	99.43	100.0	99.71	1045
Accuracy			91.77	5872
Macro Avg	92.58	91.72	91.79	5872
Weighted Avg	92.52	91.77	91.80	5872

**Table 6:** Classification Report on MobiAct (%).

Class	Precision	Recall	F1 Score	Support
Standing	94.19	99.27	96.66	6445
Walking	99.38	88.82	93.80	5964
Jogging	95.34	94.00	94.67	1718
Jumping	99.77	99.88	99.83	1736
Stairs up	70.84	87.42	78.26	906
Stairs down	68.23	90.45	77.78	838
Stand to sit	91.18	72.37	80.69	257
Sitting	91.94	95.13	93.51	863
Sit to stand	80.00	70.33	74.85	91
Car-step in	80.71	70.10	75.03	388
Car-step out	77.41	60.61	67.99	424
Accuracy			94.49	25,116
Macro Avg	84.75	84.03	84.01	25,116
Weighted Avg	94.60	94.49	94.44	25,116

**Table 7:** Classification Report on UCS-HAD (%).

Class	Precision	Recall	F1 Score	Support
Walking Forward	69.90	89.80	78.64	2054
Walking Left	88.67	67.65	76.75	1354
Walking Right	90.85	76.57	83.10	1354
Walking Upstairs	94.51	83.38	88.60	1342
Walking Downstairs	96.59	82.26	88.85	1274
Running Forward	91.12	94.64	92.85	672
Jumping Up	100.0	98.50	99.24	666
Sitting	89.77	79.33	84.23	1350
Standing	50.25	85.60	63.33	1160
Sleeping	100.0	100.0	100.0	1960
Elevator Up	37.08	34.99	36.00	886
Elevator Down	47.53	30.68	37.29	942
Accuracy			79.13	14,996
Macro Avg	79.69	76.96	77.41	14,996
Weighted Avg	81.08	79.13	79.17	14,996

**Table 8:** Macro-Average F1 Score (%) of Different Tasks.

Task	MobiAct	UCI HAR	US-HAD
Supervised Only	75.13	89.71	71.35
SupCon (Act + User)	82.81	92.22	67.14
**MultiSupConHAR**	**86.01**	**93.16**	**77.13**

Bold numbers indicate the best performance on each dataset.

**Table 9:** Macro-Average F1 Score (%) of Different Contrastive Strategies.

Method	MobiAct	UCI HAR	USC-HAD
Supervised	82.81	92.22	67.14
SimCLR	72.28	81.35	56.94
SupCon (Act Only)	78.22	89.24	71.56
SupCon (Act + User)	75.13	89.71	71.35
Multi-task (SimCLR)	80.44	91.83	73.73
Multi-task (SupCon Act Only)	82.38	92.69	74.36
**MultiSupConHAR**	**86.01**	**93.16**	**77.13**

Bold numbers indicate the best performance on each dataset.

## Data Availability

In this study, we used three publicly available datasets—MobiAct, UCI HAR, and USC-HAD—which can be accessed online. MobiAct—https://bmi.hmu.gr/the-mobifall-and-mobiact-datasets-2/. Accessed on 1 October 2022. USC-HAD—https://sipi.usc.edu/had/. Accessed on 1 October 2022. UCI HAR—https://github.com/arijitiiest/UCI-Human-Activity-Recognition?tab=readme-ov-file. Accessed on 1 October 2022. Model checkpoints can be provided upon reasonable request for research purposes.
